# Pathophysiologic mechanisms of obesity- and chronic inflammation-related genes in etiology of polycystic ovary syndrome

**DOI:** 10.22038/IJBMS.2019.14029

**Published:** 2019-12

**Authors:** Zahra Shaaban, Arezoo Khoradmehr, Amir Amiri-Yekta, Mohammad Reza Jafarzadeh Shirazi, Amin Tamadon

**Affiliations:** 1 Department of Animal Science, College of Agriculture, Shiraz University, Shiraz, Iran; 2 Research and Clinical Center for Infertility, Shahid Sadoughi University of Medical Sciences, Yazd, Iran; 3 Reproductive Biomedicine Research Center, Royan Institute, Tehran, Iran; 4 The Persian Gulf Marine Biotechnology Research Center, The Persian Gulf Biomedical Sciences Research Institute, Bushehr University of Medical Sciences, Bushehr, Iran

**Keywords:** Chronic inflammation, Gene, Obesity, Pathophysiology, Polycystic ovary syndrome

## Abstract

**Objective(s):**

One of the common heterogeneous reproductive disorders in women of childbearing age is polycystic ovary syndrome (PCOS). It is characterized by lack of fertility due to anovulatory cycles, hyperandrogenemia, polycystic ovaries, hyperinsulinemia, and obesity. Both reproductive anomalies and metabolic disorders are involved in PCOS pathology. Although the role of increased levels of androgens in initiation of PCOS is almost proven, mechanisms of PCOS pathophysiology are not clear. Here we discuss roles of altered metabolic conditions, obesity, and chronic inflammation in PCOS pathophysiology.

**Materials and Methods:**

In this review, we attempted to identify genes related to obesity and chronic inflammation aspects of PCOS and their physiological functions to explain the pathways that are regulated by these genes and can be a prominent function in PCOS predisposition. For this purpose, published articles and reviews dealing with genetic evaluation of PCOS in women in peer-reviewed journals in PubMed and Google Scholar databases were included in this review.

**Results:**

Obesity and chronic inflammation are not prominent diagnostic features of PCOS, but they play an important role in exacerbating metabolic and hyperandrogenic states. *ADIPOQ*, *FTO* *TGFβ*, and *DENND1A* as the main obesity- and chronic inflammation-related genes have roles in PCOS pathophysiology.

**Conclusion:**

It seems that genes related to obesity pathology in genomic research association, are related to metabolic aspects and body mass index in PCOS patients. Genomes have roles in chronic inflammation, followed by obesity, in the pathogenesis of PCOS.

## Introduction

Polycystic ovary syndrome (PCOS), affects 5–15% of reproductive-aged women in the world. It is one of the most prevalent endocrine abnormalities that are characterized by biochemical hyperandrogenemia, chronic anovulation, and polycystic ovaries (1). Besides, PCOS relates to other symptoms among which insulin resistance, abdominal obesity, chronic inflammation, elevated risk of metabolic syndrome, type 2 diabetes, and cardiovascular diseases are more pronounced (1). The genetic basis of PCOS by twin-family based association studies was confirmed, and accordingly, heritability of PCOS is estimated 70% (2). The genome-wide association studies (GWAS) identified 15 susceptible single nucleotide polymorphisms (SNP) in 11 loci (*LHCGR, FSHR, THADA, INSR, DENND1A, RAB5B, C9orf3, YAP1, SUMO1P1, TOX3, and HMGA2*) in PCOS Chinese women (3, 4). These loci (*LHCGR, FSHR, INSR, THADA, DENND1A, and YAP1*) are likely more important because they were replicated in European population, too (5-8). Undoubtedly, effects of environmental factors in interacting with genetic agents in creating important features, such as hyperandrogenism and insulin resistance of PCOS cannot be ignored (9). 

The lifestyle interacts with genetics in the incidence of weight excess and obesity. For instance, sisters with irregular menses and hyperandrogenism were overweight in contrast with ones who have regular cycles and hyperandrogenism. Abdominal adiposity, excess weight, and obesity are usually common among PCOS patients. Obesity plays an underscored role in pathophysiology of insulin resistance and hyperandrogenism (10). For this reason, diet modification and exercise play an important role in improving PCOS reproductive and metabolic phenotypes (11, 12). 

PCOS is also related to elevation of inflammatory indices such as increased levels of c- reactive protein, interleukins, and white blood cell count as well as oxidative stress and endothelial dysfunction, which are components of low-grade chronic inflammation (13). Adipose tissue products can play a role in inflammation creation and its exacerbation. Interleukin 6 (IL-6) secreted from adipocytes, stimulates secretion of hepatic C-reactive protein (CRP) and both of them elevate in PCOS and obese patients (14). Abdominal obesity is associated with dysregulation of sex steroid levels in PCOS, such as androgen overproduction and reduction of sex hormone-binding globulin (SHBG). The problem is that obesity has a confounding effect on the exacerbating of PCOS main traits, hyperandrogenism, and insulin resistance (15). 

Obesity influences the development or escalation of PCOS. Mother’s obesity in late pregnancy predisposes her daughter to PCOS in adulthood, but effective pathophysiologic mechanisms are not clear (16). Also, the intrauterine androgen excess may create adiposity in offspring at adulthood. Abdominal obesity can be important in ovarian or adrenal hyperandrogenism in PCOS, although, the elevated levels of androgens can contribute to abdominal fat deposition (10). Apart from the key role of hyperandrogenism in PCOS pathophysiology, obesity, inflammation, and other metabolic factors have the main role in aggravating steroidogenic abnormalities. Furthermore, genetic predisposition underlies both primary steroidogenic disorders and other exacerbating factors (10). Obesity is related to low-grade chronic inflammation, which contributes to insulin resistance by adipocytokines functions such as tumor necrosis factor alpha (TNFα) and adiponectin (17, 18). 

In this review, we attempted to identify genes related to obesity and chronic inflammation aspects of PCOS and their physiological functions to explain the pathways that are regulated by these genes and can be a prominent function in PCOS predisposition. Initially, we searched the major databases such as PubMed and Google Scholar based on gene, PCOS, obesity, inflammation, etiology, patient, and human keywords which were taken from the MeSH site. These genes are divided into two groups that are effective in obesity and chronic inflammation, respectively. Then, they are separated from each gene, their actions are described, and thus the involved physiologic pathways identified. Eventually, hypotheses associated with these findings are presented.

Even though the role of increased levels of androgens in the initiation of PCOS is almost documented and approved by most authors, mechanisms of PCOS pathophysiology are not clear. In fact, hyperandrogenism is the common loop of different hypotheses presented on PCOS etiology. These issues are explained in detail in a previous paper about the role of steroid and gonadotropin related genes in PCOS pathophysiology (19). But roles of altered metabolic conditions are prominent.

**Obesity-related genes in etiology of polycystic ovary syndrome**

Since the genetic nature of obesity is well known (20), environmental factors such as lifestyle, nutrition, and exercise also contribute to obesity. Obesity leads to insulin resistance, followed by other events that ultimately result in the occurrence of PCOS. Obesity can result from the reduced lipolytic effect of insulin in PCOS, in turn, by increased serum inflammatory mediators such as TNFα and high-sensitivity CRP (hs-CRP), leads to beta cell dysfunction and insulin resistance of PCOS. Obesity can intensify the hyperandrogenic state in PCOS because abdominal obesity alters fat-soluble androgen clearance and deposition and also exacerbates hyperandrogenism by reduction of SHBG levels.

Obesity is associated with PCOS, and between 38–88% of PCOS patients are overweight or obese (21). Obesity is accompanied by other PCOS metabolic attributes, such as insulin resistance (22). Obesity is related to insulin resistance and compensatory hyperinsulinemia (23). Central adiposity and hyperandrogenemia by reduction of natural insulin sensitizer adipokines such as adiponectin lead to the development of insulin resistance in PCOS (24). Also, both obesity and insulin resistance elevate the risk of cardiovascular and type 2 diabetes mellitus diseases (25), which are considered metabolic features of PCOS. Body mass index (BMI) of women with PCOS is usually higher than normal women (26). Several growth factors and inflammatory factors were increased in obesity and could promote ovarian androgen overproduction (23). Thus, obesity can develop insulin resistance, androgen excess, and inflammation in PCOS women.

Dyslipidemia is prevalent in almost 70% of women with PCOS (27). In addition to obesity, dyslipidemia causes insulin resistance; in contrast, insulin resistance also affects lipid metabolism and serum lipid parameters, all of which are characteristics of PCOS (28). Dyslipidemia and metabolic syndrome have a pivotal role in PCOS development but not obesity and insulin resistance (25).

**Figure 1:**
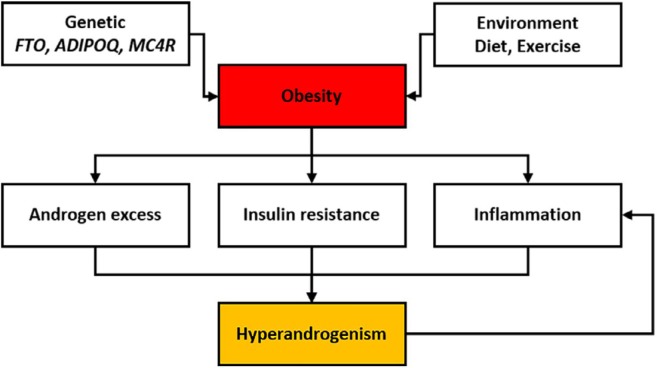
Causes of obesity and its adverse effects are expressed. Obesity mainly can occur due to genetic background and inappropriate lifestyle. Effect of abdominal obesity in exacerbating complications of PCOS is more than peripheral obesity. Abdominal obesity by alteration of metabolic clearance of androgens, increased levels of inflammatory mediators, and elevated beta cells mass in the pancreas is involved in hyperandrogenism, insulin resistance, and inflammation, respectively. The relationship between inflammation and hyperandrogenism is reciprocal, and androgens can create inflammation state

It seems that factors of genetic and lifestyle, both or alone lead to obesity in humans. Moreover, obesity has adverse effects on the physiological pathways in the body, which eventually result in PCOS metabolic parameters (Figure 1). Obesity is related to anovulation, loss of pregnancy, and occurrence of pregnancy complications (pre-eclampsia and gestational diabetes); as well as, delayed or failed responses to therapeutic strategies such as clomiphene citrate and gonadotropins. A five percent weight loss in women increased the rate of normal and spontaneous ovulation and pregnancy (25). Thus it seems the alteration of environmental factors such as exercise and diet is useful in the treatment of PCOS. But the effects of obesity-related genetic variants cannot be forgotten. Below, a number of these genes are described and are summarized in Table 1.

***ADIPOQ***

It is well known that insulin resistance is a prominent risk factor for obesity. The adipocyte cells are one of the target cells of insulin and secrete several adipokines such as adiponectin (34). About six polymorphisms of the adiponectin gene have been detected in various racial populations and with different environmental factors associated with metabolic abnormalities of PCOS. Among them, SNPs +45(T/G) and +276(G/T) are highly associated with obesity, insulin resistance, and type 2 diabetes mellitus in Asian populations (34). Also, in a family-based study of the Chinese Han population, two SNPs of the ADIPOQ gene are associated with PCOS (30). Generally, adiponectin is related to metabolic syndrome (insulin resistance, obesity, and dyslipidemia) in PCOS (42), which reflects the role of obesity in the mediation of adiponectin in PCOS. Because the genetic variants of the ADIPOQ gene in different racial populations were associated with PCOS (Table 1), this gene may be a risk factor for obesity and insulin resistance of PCOS.

***FTO***

Fat mass and obesity-associated (FTO) gene is a large gene that encodes 2-oxoglutarate-dependent nucleic acid demethylase and is mainly expressed in hypothalamic nuclei regulating feed intake. FTO has also been shown in other tissues such as liver, pancreas, muscles, and adipose tissue (43). Generally, the FTO gene is associated with type 2 diabetes mellitus and obesity (38). In studies evaluating the association of FTO and PCOS, FTO was mainly associated with BMI and anthropometric parameters in women of various races with PCOS (37-39, 44) (Table 1). Increasing evidence suggests the association of variants of FTO with hyperandrogenemia in PCOS patients (37, 38). The relationship between FTO variants and impaired glucose tolerance, insulin resistance, and hyperandrogenism, that are prominent features of PCOS, are mediated via obesity and BMI (38). So, FTO can be a main genetic factor in predisposing to PCOS, primarily via an effective role in obesity and BMI, and secondarily with influencing the metabolic parameters and hyperandrogenemia.

***MC4R***

Melanocortin-4 receptor (MC4R) gene encodes the G-protein coupled receptor that is dominantly expressed in the brain and mediates the signaling pathway of melanocortin (45). MC4R has an important role in energy homeostasis and appetite and is the main genetic cause of obesity in humans (45, 46) and animals (47-49). Due to the critical role of MC4R in obesity pathology, the association between different SNPs of the MC4R gene and BMI and obesity in PCOS patients were demonstrated (36, 39, 40). According to the evidence, the MC4R gene via a causal effect on obesity contributes to PCOS etiology. The same evidence has been shown in PCOS animal models, too (50).

***SREBP-2*** **and** ***LXR***

Given that insulin resistance disrupts lipid metabolism and serum lipid parameters, central transcription factors in metabolic pathways appear to be likely candidates for PCOS abnormalities (28). The central transcription factors of lipid metabolism, are liver X receptor (NR1H3, LXRa), and sterol regulatory element binding protein-2 (SREBP-2) (51-53). These two transcription factors regulate the expression of effective genes on lipoprotein metabolism, cholesterol homeostasis, and lipogenesis. LXRα transcription factor also plays a key action in insulin secretion of pancreatic beta cells (53). The SREBP-2, which is encoded by a single gene on human chromosome 22, also plays an important role in maintaining lipid homeostasis (54). In a case-control study on Chinese Han women, variants of these two transcription factors were associated with PCOS (28). Although more research is needed to prove this association. 

**Chronic inflammation-related genes in etiology of polycystic ovary syndrome**

Inflammation can be the key marker of endothelial dysfunction, atherosclerosis and cardiovascular diseases, and also metabolic disruptions of PCOS. It seems inflammatory reactions are more often secondary pathways in PCOS etiology and are affected by obesity and hyperglycemia. Glucose-induced nuclear factor-κB (NF-κB) activation from mononuclear cells eventually leads to beta cell dysfunction and insulin secretion irregularities in PCOS (55). Adipokines derived adipocytes contribute to inflammation and insulin resistance development. For instance, decreased adiponectin and increased TNFα and IL-6 are contributed to insulin resistance development.

PCOS is an inflammatory state, and chronic inflammatory-related genes may be effective in the incidence of PCOS through mediating role in hyperandrogenism, obesity, insulin resistance, and anovulation (56). Chronic inflammation is involved in the development of PCOS. Dietary glucose-induced oxidative stress by the particular molecular signaling pathway leads to increased production of pro-inflammatory cytokines from mononuclear cells (MNC). 

Hyperandrogenism may be the progenitor of low-grade chronic inflammation in PCOS and via increase of MNC sensitivity, stimulates glucose-induced inflammation (57). On the other hand, the pro-inflammatory cytokines, TNFα, can elevate the production of androgens with upregulation of steroidogenic enzymes and stimulation of proliferation of theca cells. Also, TNFα is a mediator of insulin resistance, thus it is likely dietary-induced inflammation, is the base of insulin resistance in PCOS (57). Exposure of androgen excess prone the adipocytes to hypertrophy and hypertrophic adipocytes were observed in PCOS women. Hypertrophic adipocytes were more predisposed to inflammation (58). Adipose tissue adipocytes in an autocrine/paracrine manner by secretion of products, some of which are inflammation factors, contributed to low-grade inflammation related to PCOS (13). The low-grade chronic inflammation in PCOS may be related to hyperandrogenism and hypertrophic adipocyte (58).

**Figure 2:**
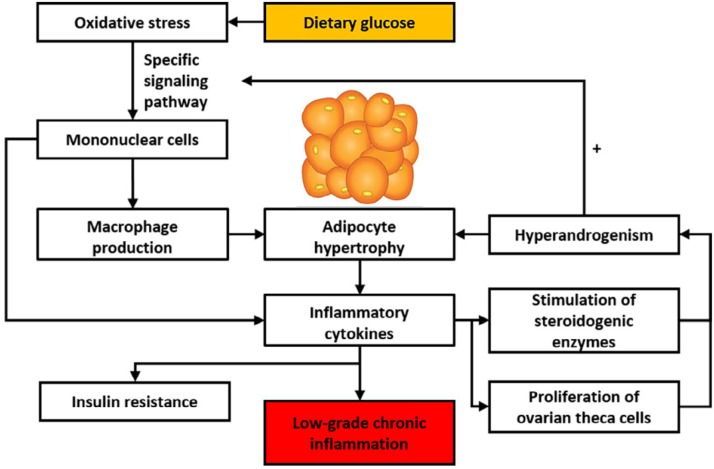
Pathophysiological pathways contribute to low-grade inflammation in polycystic ovary syndrome (PCOS). Glucose-induced oxidative stress by production of inflammatory cytokines leads to insulin resistance. Cytokines via effects on theca cells eventually create hyperandrogenism. Macrophage derived mononuclear cells resulted in adipocytes hypertrophy and in turn by secretion of inflammatory cytokine create low-grade chronic inflammation

The correlation between the incidence of PCOS and chronic inflammation has increased attention to the inflammatory factors coding genes and their association with PCOS (59). The imbalance between pro-inflammatory and anti-inflammatory cytokines and cytokine genes polymorphisms may be involved in etiology of PCOS (60). Therefore, inflammatory reactions act as mediators and contribute to the development and aggravation of metabolic properties of PCOS (Figure 2). The genes related to chronic inflammation are presented in Table 2.

***TGF-β1***

The beta-transforming growth factor-β (TGF-β1) is a component of multifunctional cytokines family and mediates wound healing, tissue fibrosis, and embryonic development (68). In recent years, pathogenic immunity factors have been widely considered in PCOS, which shows PCOS is a low-grade chronic inflammatory condition (68). PCOS patients showed higher levels of lymphocytes, monocytes, and eosinophils, plus CRPs, TNFα, and IL-6 in serum, all of which are peripheral inflammatory factors (72, 73). Moreover, PCOS ovaries showed chronic inflammation and higher numbers of inflammatory cells compared to normal conditions. Cytokines seem to be important for folliculogenesis and ovulation and also participate in the process of follicular atresia and corpus luteum regression by activating immune cells (68, 74). Polymorphism of rs4803457C/T in *TGFβ* gene was associated with susceptibility to PCOS and was demonstrated as the main constituent of PCOS development in Chinese women (68).

***TNFα***

It is reported that the TNFα cytokine is secreted by adipose tissue and plays an essential role in mediating insulin resistance and as a result of obesity (75). The TNFα pro-inflammatory cytokine is known as a mediator of insulin resistance in PCOS (57). Androgen excess promotes the release of TNFα from MNCs *in vivo* and *in vitro* (76). TNFα plays a cardinal role in oxidative stress and inflammation; in PCOS, production of TNFα is induced by hyperglycemia and hyperandrogenemia (77). Serine phosphorylation of Insulin receptor substrate 1 (IRS-1) seems to be a mechanism for insulin resistance by mediating TNFα (78). Overexpression of TNFα in peripheral tissues such as muscle and adipose, by reducing the tyrosine kinase activity in the insulin signaling pathway, is one of the mechanisms for insulin resistance development mediated by TNFα (78). Also, TNFα directly effects ovary and adrenal function (10) and then increases steroidogenesis in theca cells *in vitro* (79). Despite the finding that TNFα is the mediator of insulin resistance, a significant association of TNFα gene with PCOS was not observed.

***TNFRS1β***

The TNF receptor superfamily 1β is a member of TNFα receptors that are said to localize on the surface of all normal and tumor cells (80). Type 2 TNF receptor mediates most of the metabolic effects of TNFα. Its serum levels increase in obese subjects correlating with insulin resistance indices (81). The M196R variant in exon 6 of the TNF receptor superfamily 1β (*TNFRS1β*) gene was associated with hyperandrogenism and PCOS in the study of hyperandrogenic and PCOS Spanish women (82). It has also been suggested that TNFα cytokines were able to induce proliferation of theca internal cells in porcine ovaries (80).

***IL-6***

IL-6 is a pleiotropic cytokine and is secreted by numerous cells including lymphocytes, monocytes, and endothelial cells. Interleukin-6 is involved in reproductive physiologic processes, such as regulating of ovarian steroid production, follicular maturation, fertilization, implantation, and modulating of ovarian development and functions (70). Concentrations of the IL-6 and CRP increase in obese women, but not in PCOS patients (14). It is shown that the polymorphism of the promoter region of the *IL-6* gene could be associated with the occurrence of metabolic abnormalities in Turkish PCOS women (60). Also, IL-6 may have a direct effect on ovaries and adrenal cells (10). *In vitro* studies have shown that IL-6 induced the function of human adrenal cells, in turn, elevated steroidogenesis in adrenals (83). The results of an investigation of *IL-6* gene polymorphisms are very inconsistent (Table 2), which is possibly related to racial background, genetic variants, and epigenetic environmental factors among different populations (70).

***DENND1A***

Differentially expressed in normal and neoplastic (cell) domain containing 1A (DENND1A) is a member of connecdenn proteins family. These proteins have differential expression in normal and neoplastic domains of cells (84). The *DENND1A* gene encodes the connecdenn-1 protein, which has a clathrin-binding domain and facilitates receptor-mediated endocytosis and participates in endosomes trafficking (84, 85). Overexpression of variant 2 DENND1A, alternative splicing of DENND1A, in PCOS theca cells was observed, which can contribute to androgen overproduction in these cells (86). High expression of variant 2 DENND1A leads to over mRNA expression of cytochrome P450 17A1 (*CYP17A1*) and hyperactivity of CYP11A1 and CYP17A1, which in turn increases the level of androgen production in theca cells (86). So, DENND1A can be a mediator for hyperandrogenism of PCOS, and maybe through this pathway connect to hyperandrogenism, the common loop of all hypotheses of PCOS. Previous studies reported DENND1A as a susceptibility locus in PCOS, but there are racial differences about this association (64). Even though the DENND1A locus in various GWAS among different populations is identified as a risk locus in PCOS and maybe a valuable gene in PCOS pathogenesis and diagnosis, it is not applicable to all populations.

**Table1:** Candidate genes involved in etiology of polycystic ovary syndrome related to obesity and dyslipidemia

**Gene**	**Genetic marker(s)**	**Type of study**	**Physiologic function**	**Studied population**	**Type of polymorphism**	**References**
**ADIPOQ**	T45G G276T	Meta-analysis	Glucose regulation and fatty acid oxidation	Different population	G276T	(29)
**ADIPOQ**	rs2241766 rs1501299	Family-based analysis	Glucose regulation and fatty acid oxidation	Chinese Han	rs1501299	(30)
**ADIPOQ**	SNPs at position −11377 of the ADIPOQ gene	Case-control	Glucose regulation and fatty acid oxidation	Japanese	ND	(31)
**ADIPOQ**	45T/G 276G/T	Meta-analysis	Glucose regulation and fatty acid oxidation	Different population	45T/G 276G/T	(32)
**ADIPOQ**	C45G15G(T/G) C276(G/T)	Case-control	Glucose regulation and fatty acid oxidation	Chinese Han	+45G15G(T/G) +276(G/T)	(33)
**ADIPOQ**	+45G15G(T/G) +276(G/T)	Case-control	Glucose regulation and fatty acid oxidation	Korean	+276(G/T)	(34)
**FTO**	rs9939609	Case-control	Energy homeostasis regulation	British/Irish	rs9939609	(35)
**FTO**	Five SNPs	Family-based and case-control	Energy homeostasis regulation	White American	rs1421085	(36)
**FTO**	rs9939609	Case-control	Energy homeostasis regulation	Australian	rs9939609	(37)
**FTO**	rs1421085 rs17817449 rs8050136	Case-control	Energy homeostasis regulation	Korean	rs1421085 rs17817449 rs8050136	(38)
**MC4R**	rs17782313 (T/C)	Case-control	Regulator of melanocortin neuronal pathways	Chinese	rs17782313	(39)
**MC4R**	rs12970134	Case-control	Regulator of melanocortin neuronal pathways	Czech	rs12970134	(40)
**ADIPOQ**	rs2241766	Case-control	Metabolic features of PCOS	Iranian	rs2241766 “TT”	(41)
**SREBP-2 LXRa**	rs2228314 rs11039155	Case-control	Lipid metabolism	Chinese Han	rs2228314 G to C rs11039155 G to A	(28)

*ADIPOQ*: Adiponectin; *FTO*: Fat mass, and obesity-associated; *MC4R*: Melanocortin 4 receptor; *SREBP-2*: Sterol Regulatory Element Binding Protein-2; *LXRa*: Liver X Receptor a; ND: No data

**Table 2:** Candidate genes involved in etiology of polycystic ovary syndrome related to cell proliferation and signaling and chronic inflammation

**Gene**	**Genetic marker(s)**	**Type of study**	**Physiologic function**	**Studied population**	**Type of polymorphism**	**Reference**
**DENND1A**	rs2479106	Case control	Clathrin-mediated endocytosis	Caucasian	rs2479106 G	(61)
**DENND1A**	rs10818854 rs2479106 rs10986105	Meta-analysis	Clathrin-mediated endocytosis	Asian and European	rs10818854 rs10986105	(62)
**DENND1A**	rs2479106 rs10818854	Meta-analysis	Clathrin-mediated endocytosis	Chinese Han	rs10818854	(63)
**DENND1A**	rs10818854 rs2479106 rs10986105	Retrospective case-control study.	Clathrin-mediated endocytosis	Tunisian	0818854 rs10986105	(64)
**AQP8**	rs7198838 rs1076973 rs1076974 rs2287797 rs2287798 rs2287796	case control	Water channel protein	Chinese Han	rs2287798	(65)
**YAP1 **	rs11225138 rs11225161 rs1122516	Replication study	Transcriptional regulator	Chinese Han	rs11225161 (A/G)	(66)
**TNF**	rs1799964 rs1799724	Family association	Pro-inflammatory cytokine	Chinese Han	rs1799964	(67)
**TGF-β1**	rs4803457C/T rs11466313 deletion/AGG rs2217130C/T rs1800469C/T rs1800470C/T	GWAS	Low-grade chronic inflammation	Chinese Han	rs4803457C/T	(68)
**TNFα** **IL-6** **IL-10**	−308 G/A −174 G/C −1082 G/A	case-control	Inflammatory cytokines	Turkish	IL-6 promoter region polymorphism	(60)
**TNFRSF1B**	M196R (676 T→G) variant in exon 6	GWAS	TNF signaling	Spanish Italian	M196R (676 T→G)	(69)
**IL-6**	-174 G/C	Case- control	Inflammatory cytokine	Indian	−174 G/C SNP	(70)
**TNFα** **IL-6**	(−308 G/A), (−174 G/C)	meta-analysis	Chronic low-grade inflammation	Different populations	No association	(71)

*DENND1A*: DENN domain containing 1A; *AQP8*: Aquaporin 8; *YAP1*: yeast associated protein 1; *TNF*: Tumor necrosis factor; *TGF-β1*: transforming growth factor β1; *TNFRSF1B*: TNF receptor 2

## Conclusion

To sum up, identifying the root cause of PCOS as a heterogeneous disorder is hard and at the same time, it provides the basis of many investigations. So far, the role of hyperandrogenism and its pre- and post-pathways in PCOS development is better explained and confirmed; various backgrounds including genetic, environmental factors, and developmental origins can interfere in its creation. Hyperandrogenism can also be caused by insulin resistance. While the roles of hyperandrogenism and hyperinsulinemia are the major reasons for PCOS development, but abdominal obesity and low-grade inflammation can play a significant role through the mediation of some pathways leading to insulin resistance and hyperandrogenism. As foregoing, chronic inflammation and obesity are not necessarily primary factors, and sometimes their role exacerbates the syndrome or even themselves as a consequence of the syndrome. It seems that genes related to obesity pathology (*FTO, ADIPOQ*, and *MC4R*), in genomic research association, are related to metabolic aspects and BMI in PCOS patients. These findings suggest that obesity, especially abdominal phenotype, completes and exacerbates the phenotypic picture of PCOS. Inflammation also occurs, followed by obesity and has the same role in PCOS. It should be kept in mind that these hypotheses are better evaluated by clinical and experimental research such as animal models and therapeutic approaches. 
